# Higher 25(OH)D Levels at Admission Predict a Favorable Disease Evolution in Moderate-to-Severe COVID-19 Patients

**DOI:** 10.3390/ijms27083541

**Published:** 2026-04-16

**Authors:** Manuela Rizzi, Federica Vincenzi, Stelvio Tonello, Eleonora Rizzi, Giuseppe Francesco Casciaro, Erica Matino, Martina Costanzo, Erika Zecca, Alessandro Croce, Anita Pedrinelli, Veronica Vassia, Raffaella Landi, Iris Zeqaj, Francesca Boccafoschi, Paolo Amedeo Tillio, Roberta Rolla, Umberto Dianzani, Luigi Mario Castello, Mario Pirisi, Donato Colangelo, Pier Paolo Sainaghi

**Affiliations:** 1 Dipartimento di Scienze della Salute (DiSS), Università del Piemonte Orientale (UPO), 28100 Novara, Italyroberta.rolla@med.uniupo.it (R.R.); donato.colangelo@med.uniupo.it (D.C.); 2 Dipartimento di Medicina Traslazionale (DiMeT), Università del Piemonte Orientale (UPO), 28100 Novara, Italy; federica.vincenzi@uniupo.it (F.V.);; 3 Internal Medicine and Rheumatology Department, Azienda Ospedaliero-Universitaria “Maggiore della Carità” di Novara, 28100 Novara, Italy; eleonora.rizzi@maggioreosp.novara.it (E.R.); veronica.vassia@gmail.com (V.V.);; 4 Clinical Biochemistry Laboratory, Azienda Ospedaliero-Universitaria “Maggiore della Carità” di Novara, 28100 Novara, Italy; 5 IRCAD (Interdisciplinary Research Center of Autoimmune Diseases), Università del Piemonte Orientale (UPO), 28100 Novara, Italy; 6 CAAD (Center for Translational Research on Allergic and Autoimmune Diseases), Università del Piemonte Orientale (UPO), 28100 Novara, Italy; 7 Internal Medicine Unit, Azienda Ospedaliero-Universitaria “SS. Antonio e Biagio e Cesare Arrigo”, 15100 Alessandria, Italy

**Keywords:** COVID-19, vitamin D, biomarker, clinical outcomes

## Abstract

Research into effective predictive markers and therapeutic interventions for COVID-19 remains of considerable interest. Vitamin D may be relevant, especially in frail populations in whom deficiency is more prevalent. In this prospective observational cohort study, 139 patients with moderate-to-severe COVID-19 who were hospitalized during the third wave of the pandemic in Italy were enrolled. Plasma vitamin D concentrations (both 25-hydroxyvitamin D-25(OH)D and 1,25-dihydroxyvitamin D-1,25(OH)_2_D) together with parathyroid hormone levels were measured using a chemiluminescent assay validated for clinical use on automated laboratory platforms. Plasma vitamin D levels were below the sufficiency threshold. Notably, 25(OH)D concentrations were significantly lower in patients who experienced a negative outcome (11.10 [8.80–16.20] vs. 15.25 [9.90–24.80] ng/mL, *p* = 0.0450) and significantly higher in patients with rapid clinical recovery (15.25 [10.70–24.80] vs. 13.30 [7.47–19.60] ng/mL, *p* = 0.0446) compared with all other patients. Through multivariable logistic regression analysis, higher 25(OH)D levels at the time of hospitalization were confirmed as an independent predictor of favorable outcome. A plasma 25(OH)D concentration above 11.10 ng/mL predicted favorable disease resolution, with a positive likelihood ratio of 1.40 (IQR: 1.05–1.87). In conclusion, our findings support plasma vitamin D levels as an independent predictor of clinical outcomes in patients hospitalized with COVID-19 pneumonia.

## 1. Introduction

Severe acute respiratory syndrome coronavirus 2 (SARS-CoV-2), the etiological agent of coronavirus disease 2019 (COVID-19), was first identified in December 2019, following an outbreak of pneumonia of unknown origin in Wuhan district, China. Although the World Health Organization (WHO) Emergency Committee declared the end of the public health emergency of international concern in mid-2023 [1], SARS-CoV-2 continues to circulate globally. Ongoing viral activity, with an estimated positivity rate of approximately 11% reported by the Global Influenza Surveillance and Response System (GISRS), underscores the need for continued surveillance of this evolving situation [2].

It is now well recognized that COVID-19 is a highly heterogeneous disease, ranging from asymptomatic or mildly symptomatic infection to life-threatening clinical manifestations as interstitial pneumonia, acute respiratory distress syndrome (ARDS), and multiorgan failure [3,4,5]. COVID-19 has been described as the most significant pandemic of the past century [6]. Although the widespread availability of anti-COVID-19 vaccines and the implementation of early targeted therapies have substantially reduced the incidence of severe disease, SARS-CoV-2, characterized by a high mutation rate, remains a major public health concern. This is particularly relevant for unvaccinated or under-vaccinated individuals, as well as for elderly patients and those with multiple comorbidities [7,8,9,10,11]. Notably, severe COVID-19 is frequently associated with hypercytokinemia, dysregulated and aberrant immune responses, and coagulation abnormalities, all of which contribute to organ dysfunction and clinical deterioration [12]. Since the early phases of the pandemic, various biomarkers have been investigated for their potential to predict disease progression at disease onset, thereby supporting risk stratification and clinical decision-making [13,14].

Among the non-classical biomarkers evaluated in patients with COVID-19, vitamin D has attracted growing interest. Beyond its established role in mineral homeostasis, vitamin D exerts multiple pleiotropic biological effects. It is recognized as an important modulator of both innate and adaptive immune responses, with the ability to downregulate pro-inflammatory cytokine production and enhance antiviral defense mechanisms [12,15,16,17,18]. Advanced age, impaired immune function (including immunosuppressive or immunodeficiency states), and the presence of comorbidities are among the most relevant risk factors for severe COVID-19. These conditions are frequently associated with hypovitaminosis D, which has been linked to increased susceptibility to respiratory tract infections, including SARS-CoV-2 infection [12]. Given the high prevalence of vitamin D insufficiency in Western countries [17,19], the present prospective cohort study aimed to assess circulating vitamin D levels in patients with moderate-to-severe COVID-19 admitted to non-intensive care units (non-ICUs) of a tertiary care hospital in Northern Italy during the third pandemic wave, in order to evaluate their potential role in predicting disease evolution.

## 2. Results

We analyzed 139 available samples from consecutive SARS-CoV-2-positive patients hospitalized in non-ICU wards at the Maggiore della Carità University Hospital, enrolled in the BIAS cohort between January and May 2021.

The study population showed a slight male predominance (87 males vs. 52 females; 62.6% vs. 37.4%). Regarding treatments administered prior to hospital admission, the most frequently reported therapies were enoxaparin (43 patients, 30.9%) and corticosteroids (72 patients, 51.8%). At the time of hospital admission, 9 patients (6.5%) presented with severe respiratory failure (defined as PaO_2_/FiO_2_ < 100), 102 patients with moderate respiratory failure (defined as 100 ≤ PaO_2_/FiO_2_ < 200), while 28 patients showed mild or no respiratory failure (PaO_2_/FiO_2_ ≥ 200). Consistent with this clinical presentation, the median NEWS2 score at admission was 5 (IQR: 4–6). A detailed description of the study cohort is reported in Table 1.

Among the 139 patients included in the study, 30 (21.6%) experienced an adverse in-hospital outcome, defined as death during hospitalization or transfer to the intensive care unit (ICU), while 90 patients (64.7%) achieved a favorable clinical outcome, defined as discharge alive and/or a NEWS2 score ≤2 maintained for at least 24 h within the first 14 days of hospitalization.

The association between plasma vitamin D levels at admission (assessed as 25-hydroxyvitamin D-25(OH)D and 1,25-dihydroxyvitamin D-1,25(OH)D) and parathyroid hormone (PTH) concentrations and the predefined clinical endpoints was evaluated by univariate analysis. As reported in Table 2, admission 25(OH)D levels were significantly lower in patients experiencing an adverse outcome (death or ICU admission) compared with all other patients (11.10 ng/mL vs. 15.50 ng/mL; *p* < 0.05). Conversely, both 25(OH)D and 1,25(OH)D levels at hospital admission were significantly higher in patients with a favorable disease course, defined as discharge alive and/or a NEWS2 score ≤2 for at least 24 h within 14 days from hospital admission, compared with all other patients (15.25 ng/mL vs. 13.30 ng/mL and 73.70 ng/mL vs. 66.20 ng/mL, respectively; *p* < 0.05) (Table 3).

As baseline 25-hydroxyvitamin D levels were the only parameter showing a statistically significant association with both predefined endpoints, the robustness of this variable as a predictor of clinical disease evolution was further assessed using stepwise logistic regression analysis. As reported in Table 4, baseline 25(OH)D levels did not retain independent predictive value for the adverse endpoint. In contrast, 25(OH)D remained a significant predictor of the favorable endpoint in the multivariable model (Table 5).

**Figure 1:**
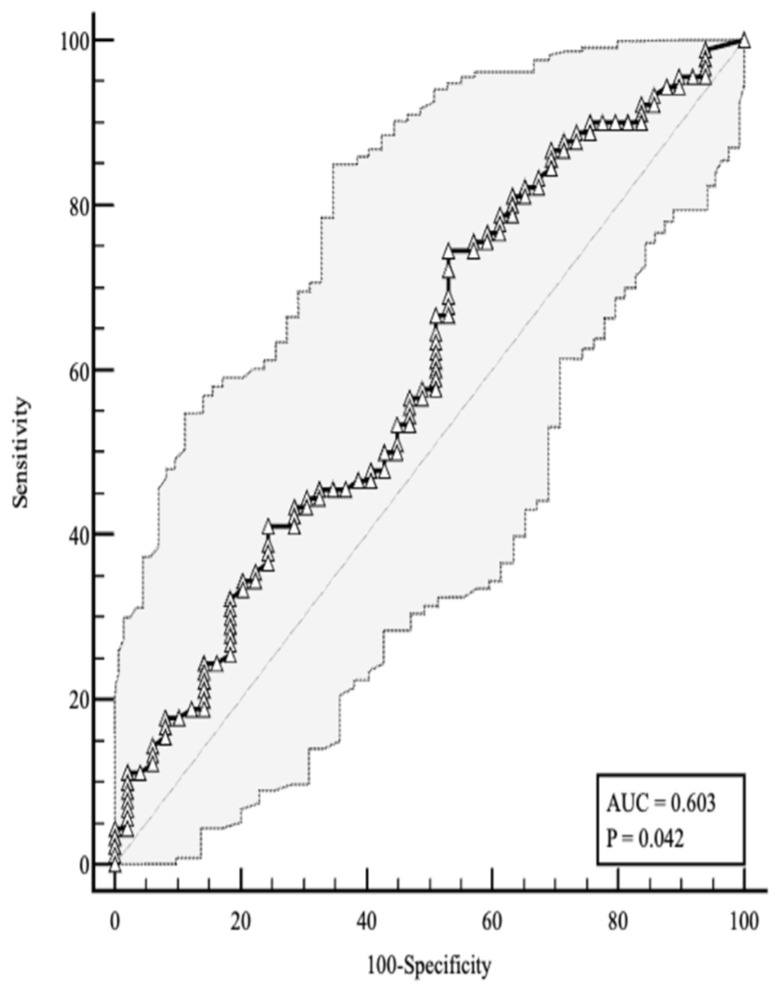
ROC curve for plasma 25(OH)D levels predicting the positive endpoint.

The accuracy of baseline 25-hydroxyvitamin D levels in predicting the favorable endpoint was assessed by receiver operating characteristic (ROC) curve analysis. As shown in Figure 1, a 25(OH)D concentration at hospital admission >11.10 ng/mL yielded a sensitivity of 74.44% and a specificity of 46.94% for predicting a favorable clinical outcome, with a positive likelihood ratio of 1.40 (IQR: 1.05–1.87).

To enhance the clinical interpretability of the identified plasma vitamin D threshold as a predictor of survival in patients with moderate-to-severe SARS-CoV-2 infection requiring hospitalization, the multivariable model was re-evaluated using a baseline plasma 25(OH)D cut-off value of 11 ng/mL (Table 6).

## 3. Discussion

Although the COVID-19 pandemic emergency ended in mid-2023 [1], several SARS-CoV-2 variants continue to circulate worldwide [20], underscoring the need for continuous updates of current knowledge on COVID-19 pathogenesis, disease progression, and long-term sequelae.

Since the beginning of the COVID-19 pandemic, ageing has been recognized as a major risk factor for the development of severe disease manifestations [21,22]. In parallel, ageing is well known to be associated with an increased prevalence of hypovitaminosis D, a condition that in older populations may result from reduced sunlight exposure as well as decreased dietary intake and intestinal absorption [23,24,25]. Given the widely recognized immunomodulatory effects of vitamin D on both innate and adaptive immune responses, as well as its role in the regulation of inflammatory pathways, it is therefore not surprising that vitamin D status has attracted considerable interest in the context of COVID-19 [15,26,27].

This prospective observational study enabled the assessment of plasma vitamin D levels at the time of hospital admission in a cohort of patients hospitalized in non-ICU wards for moderate-to-severe COVID-19 during the third wave of the pandemic in Italy. In this context, a key strength of the present study is the evaluation of vitamin D status at a very early stage of hospitalization, within 24 h of admission, thus minimizing the potential confounding effect of in-hospital interventions or disease progression on vitamin D levels. This temporal proximity to clinical presentation supports the interpretation of baseline 25(OH)D concentrations as a prognostic biomarker rather than a mere epiphenomenon of severe disease. As all enrolled patients underwent blood sampling within 24 h of hospital admission, it was possible to evaluate their baseline vitamin D status, as well as to characterize their cytokine and chemokine profiles [5].

According to the results, patients experiencing the most severe disease manifestations—leading to in-hospital death or ICU admission—not only exhibited an altered cytokine and chemokine profile [5], but also had lower plasma vitamin D levels compared with patients showing a favorable disease course.

Although the entire study cohort exhibited hypovitaminosis D, defined as a plasma 25-hydroxyvitamin D concentration <20 ng/mL [15], it is noteworthy that the 30 patients who reached the adverse study endpoint (in-hospital death or ICU admission) presented with markedly lower 25(OH)D levels at admission (11.10 ng/mL), consistent with severe hypovitaminosis D. This pronounced deficiency may have contributed to the progressive clinical deterioration observed in these patients. Importantly, the identified 25(OH)D threshold associated with favorable outcome (approximately 11 ng/mL) is markedly lower than the conventional sufficiency cut-off, suggesting that even modest differences within a range of severe hypovitaminosis D may have relevant clinical implications in the acute inflammatory setting of COVID-19. This observation aligns with previous reports indicating that relative, rather than absolute, vitamin D deficiency may influence short-term outcomes during severe infections.

Conversely, although still below the sufficiency threshold, plasma vitamin D levels at admission were higher in patients with a favorable disease course—defined as discharge alive or maintenance of a NEWS2 score ≤2 for at least 24 h within the first 14 days of hospitalization—than in those with an adverse outcome.

In this context it could be speculated that basal vitamin D levels may act as a surrogate indicator of patient’s underlying immune and inflammatory status at the time of infection, with higher basal 25(OH)D accounting for a better-regulated immune response, finally improving patient ability to counteract the dysfunctional immune response commonly observed in moderate-to-severe COVID-19 patients. Of note, only 25(OH)D levels—which represent the most commonly assessed form of vitamin D, owing to their longer half-life compared with 1,25(OH)D—showed prognostic value for both study endpoints. The lack of statistical significance of 1,25(OH)D with respect to the adverse endpoint may be attributable to the limited number of samples available from patients with an unfavorable disease course. Given that the p value obtained in the univariate analysis was close to the threshold for statistical significance, it may be speculated that in a larger cohort of patients experiencing an adverse outcome this association could also reach statistical significance, as observed for the positive endpoint. Furthermore, it should be acknowledged that the observed lack of statistical significance for 1,25(OH)D with respect to the negative endpoint could be ascribed also to the level of this compound in each target tissue. According to that, one of the key aspects of vitamin D’s biological action is represented by the level of 25(OH)D that could be reached in each target tissue, where it will undergo the conversion to the active 1,25(OH)D form, which will modulate the specific tissue responses. As a matter of fact, it is well-recognized that vitamin D intracellular levels does not necessarily correlate with the measured plasmatic levels. Finally, it should also be considered that from a pathophysiological perspective, circulating 25(OH)D represents the most reliable marker of vitamin D status, as its levels reflect body stores and are less susceptible to acute fluctuations driven by inflammation, renal function or parathyroid hormone regulation. In contrast, 1,25(OH)D concentrations are tightly regulated and may not accurately capture vitamin D availability at the tissue level during acute systemic illness.

Considering that the existing literature strongly supports the regulatory role of vitamin D in inflammation—by reducing the production of pro-inflammatory cytokines (i.e., IL-6 and TNF-α) and increasing that of anti-inflammatory cytokines (i.e., IL-10) [28,29], the correlation between baseline vitamin D status and COVID-19 clinical course observed in the univariate analysis was further explored using multivariate models. Following stepwise logistic regression analysis incorporating both baseline vitamin D levels and demographic variables, 25(OH)D lost its predictive value for the adverse endpoint, while retaining its predictive ability for a favorable disease course.

The present findings are consistent with the existing literature which, despite some conflicting results, supports an association between baseline vitamin D status and the risk of SARS-CoV-2 infection and/or COVID-19 outcomes [15,30,31,32]. The beneficial effects of vitamin D on overall health are well recognized; accordingly, several clinical trials have been conducted in recent years to evaluate the efficacy of vitamin D supplementation in improving COVID-19 outcomes. However, these studies have yielded inconclusive results, with some reporting beneficial effects while others failed to demonstrate significant improvements in clinical outcomes [33,34,35]. This heterogeneity underscores the need for well-designed, adequately powered randomized controlled trials to inform evidence-based clinical guidelines for vitamin D supplementation in the management of COVID-19.

The present study has both limitations and strengths. The main limitations relate to the study design; specifically, its single-center nature limits the generalizability of the findings. Moreover, as the study was conducted in a real-world clinical setting and vitamin D is not routinely assessed during COVID-19 hospitalization, information regarding patients’ prior vitamin D supplementation may have been incomplete. Conversely, a key strength of the study lies in the comprehensive assessment of vitamin D status, including measurements of both 25(OH)D (the most commonly evaluated form) and 1,25(OH)D, in conjunction with parathyroid hormone levels. Given that alterations in PTH concentrations are known to occur in systemic inflammatory syndromes [36,37], this combined approach provides a more comprehensive characterization of hypovitaminosis D in the study cohort.

## 4. Materials and Methods

### 4.1. Patients

In the context of the multicenter observational study “BIAS” (Baseline immunity status effect on SARS-CoV-2 presentation and evolution: comparison between immunocompetent and immunocompromised patients; local Ethics Committee approval CE7/21), between January and May 2021 (corresponding to the third wave of COVID-19 in Italy), 139 consecutive patients admitted to non-intensive care wards (including high-dependency/sub-intensive units) at the Maggiore della Carità University Hospital in Novara, Italy, were enrolled in this prospective observational cohort study. Patients were enrolled according to predefined inclusion and exclusion criteria and were required to date and sign a written informed consent form before undergoing any study-specific procedures. Inclusion criteria were: age ≥18 years; need for hospitalization due to COVID-19; SARS-CoV-2 infection confirmed by molecular (RT-PCR) or third-generation antigen testing; and symptom onset within the previous 12 days. Patients with extremely critical clinical conditions (requiring immediate admission to the intensive care unit or presenting with signs of imminent death), as well as those with end-stage (stage V) renal failure or with oncological diseases not amenable to medical or surgical treatment, were excluded. For all eligible patients, study enrolment occurred within 24 h of hospital admission, and standard treatment was initiated immediately according to local institutional protocols (including oxygen supplementation, corticosteroids, and low-molecular-weight heparin (LMWH), unless contraindicated), irrespective of the patient’s decision to participate in the study.

### 4.2. Endpoints Definition

The primary endpoints were defined as follows:

(1) The association between baseline plasma vitamin D levels and an adverse in-hospital outcome (in-hospital death or ICU admission at any time during hospitalization);
(2) The association between baseline plasma vitamin D levels and a favorable in-hospital outcome, defined as discharge from hospital within 14 days from admission and/or a National Early Warning Score 2 (NEWS2) ≤2 maintained for at least 24 h within the first 14 days of hospitalization.

### 4.3. Blood Sample Collection

Blood samples for routine laboratory analyses and vitamin D quantification were collected by drawing blood at the time of hospital admission. Immediately after collection, samples were centrifuged to obtain the relevant fractions, which were stored at −80 °C until analysis.

### 4.4. Routine Laboratory Evaluation

All patients underwent routine laboratory testing, including complete blood count and standard biochemical parameters (i.e., creatinine, alanine aminotransferase—ALT, aspartate aminotransferase—AST), as well as specific biomarkers of inflammation (i.e., C-reactive protein—CRP, interleukin-6—IL-6), hypercoagulable state (i.e., D-dimer), and iron overload (i.e., ferritin).

### 4.5. Vitamin D Evaluation

Plasma vitamin D concentrations (25-hydroxyvitamin D-25(OH)D and 1,25-dihydroxyvitamin D-1,25(OH)D) were measured at the Clinical Biochemistry Laboratory of Maggiore della Carità University Hospital (Novara, Italy) using commercially available chemiluminescence assays validated for clinical use (LIAISON^®^ 25 OH Vitamin D Total, product no. 310600, and LIAISON^®^ XL 1,25-Dihydroxyvitamin D, product no. 310980; DiaSorin SpA, Saluggia, Italy).

### 4.6. Parathyroid Hormone Evaluation

Plasma parathyroid hormone (PTH) concentrations were measured at the Clinical Biochemistry Laboratory of Maggiore della Carità University Hospital (Novara, Italy) using commercially available chemiluminescence assays validated for clinical use (LIAISON^®^ PTH, product no.310630; DiaSorin SpA, Saluggia, Italy).

### 4.7. Data Collection and Statistical Analysis

Relevant patient data (demographic characteristics, clinical variables, therapeutic regimens, and laboratory parameters) were collected and managed using the web-based REDCap database platform. Clinical and routine laboratory data were retrieved through detailed review of medical records from hospital admission (baseline, t0) until discharge (or up to a maximum follow-up of 28 days) or study exit (death or ICU admission). Data extracted from the REDCap platform, together with vitamin D measurements, were analyzed using univariate and multivariate statistical methods to assess their association with the predefined endpoints. Continuous variables were described using measures of central tendency and dispersion (median and interquartile range—IQR), while categorical variables were reported as frequencies and percentages. Odds ratios (ORs) were calculated with 95% confidence intervals (CIs). The choice of statistical tests was based on data distribution and variable type. Variables showing significance in univariate analyses were included in multivariable regression models. Receiver operating characteristic (ROC) curve analyses were performed to identify prognostic cut-off values for vitamin D levels, based on the corresponding area under the curve (AUC). Statistical significance was set at a two-tailed *p* value <0.05. Statistical analyses were conducted using Statistica for Windows, version 12 (TIBCO Software Inc., Palo Alto, CA, USA) and MedCalc^®^ statistical software, version 20.014 (MedCalc Software Ltd., Ostend, Belgium).

## 5. Conclusions

Data from this prospective observational cohort of patients with moderate-to-severe COVID-19 support the measurement of vitamin D levels at hospital admission as a potential aid in predicting disease course. Specifically, this study indicates that a baseline plasma 25(OH)D concentration above 11.10 ng/mL independently predicts a more favorable clinical course, ultimately resulting in discharge alive or maintenance of a NEWS2 score ≤2 for at least 24 h within 14 days of hospital admission.

The proposed cut-off value of 11.10 ng/mL could be considered an early biomarker of frailty, which may assist clinicians in refining the identification of patients who could benefit from closer monitoring and/or more intensive therapeutic management.

It should be emphasized that the present findings do not support vitamin D measurement as a stand-alone decision-making tool, but rather as a complementary biomarker to be interpreted in conjunction with established clinical and laboratory parameters. From this perspective, baseline 25(OH)D assessment may contribute to early risk stratification without implying causality or direct therapeutic indications.

Furthermore, given the broad range of beneficial effects associated with vitamin D repletion, well-designed interventional clinical trials are warranted to determine the most effective supplementation regimens for improving outcomes in patients with moderate-to-severe COVID-19. This is because even a small increase in basal vitamin D level could be a clinical determinant driving disease evolution, as demonstrated by the faster recovery observed in patients with higher 25(OH)D levels at hospital admission.

## Figures and Tables

**Table 1:** Baseline demographic, clinical, and laboratory characteristics of the study population. ^§^ refers to data obtained with oxygen supplementation.

	Median	IQR
Age (years)	64.23	56.19–72.00
Days from illness onset and hospital admission	6	5–8
Clinical scores		
Charlson Comorbidity Index	2	1–3
NEWS2	5	4–6
Vital parameters		
Temperature (°C)	36.50	36.10–36.70
Heart rate (beats/min)	85	75–95
Respiratory rate (breaths/min) ^§^	22	18–26
SpO_2_ ^§^	96.00	94.00–98.00
Laboratory findings		
Hemoglobin (g/dL)	14.20	12.50–15.00
Leukocytes (cells × 10^3^/μL)	6.99	5.05–9.70
Neutrophils (cells × 10^3^/μL)	5.66	4.20–8.56
Eosinophils (cells × 10^3^/μL)	0.00	0.00–0.00
Lymphocytes (cells × 10^3^/μL)	0.70	0.53–0.95
Platelets (cells × 10^3^/μL)	205.00	161.00–263.00
ALT (U/L)	37.00	28.00–55.00
AST (U/L)	41.00	31.00–57.00
Bilirubin (mg/dL)	0.60	0.50–0.80
Creatinine (mg/dL)	0.81	0.64–0.96
Glomerular filtration rate (mL/min)	90.00	70.00–103.00
CRP (mg/dL)	8.00	4.40–12.97
LDH (U/L)	718.00	539.00–870.00
Troponin I (ng/mL)	8.00	3.00–15.00
ESR (mm/h)	40.00	27.00–51.00
Ferritin (ng/mL)	820.50	361.50–1340.00
D-dimer (μg/mL)	697.00	517.00–1317.00
IL-6 (pg/mL)	11.30	5.00–31.30
Albumin (g/dL)	4.00	3.60–4.20

**Table 2:** 25(OH)D, 1,25(OH)D and parathyroid hormone association with the negative endpoint, defined as an adverse disease outcome (death or ICU admission). For each category, the number of valid entries has been indicated (*n*). Bold indicates statistically significant values.

Variable	Adverse Disease Outcome			All Other Patients			Z	*p*-Value
	*n*	Median (ng/mL)	IQR	*n*	Median (ng/mL)	IQR		
PTH	29	21.10	14.50–32.80	106	19.60	11.60–29.30	−0.8681	0.3854
25(OH)D	30	11.10	8.80–16.20	109	15.50	9.90–24.80	2.0045	**0.0450**
1,25(OH)D	27	64.00	34.80–83.00	94	72.15	52.70–102.00	1.8770	0.0605

**Table 3:** 25(OH)D, 1,25(OH)D and parathyroid hormone association with the positive endpoint, defined as a positive disease outcome (discharge alive and/or NEWS2 ≤ 2 for at least 24 h within 14 days from hospital admission). For each category, the number of valid entries has been indicated (*n*). Bold indicates statistically significant values.

Variable	Positive Disease Outcome			All Other Patients			Z	*p*-Value
	*n*	Median (ng/mL)	IQR	*n*	Median (ng/mL)	IQR		
PTH	87	19.90	11.50–29.30	48	19.35	14.70–31.65	0.8045	0.4211
25(OH)D	90	15.25	10.70–24.80	49	13.30	7.47–19.60	−2.0082	**0.0446**
1,25(OH)D	76	73.70	52.10–104.50	45	66.20	52.70–80.90	−2.0406	**0.0413**

**Table 4:** Stepwise logistic regression of demographic and clinical predictors of an adverse disease outcome (death or ICU admission). The variables entered into the model are shown in the table. 25(OH)D and NEWS2 score did not enter in the model. Bold indicates statistically significant values.

Parameter	Coefficient	*p*-Value	Odds Ratio	95% CI
Age	0.0754	**0.0007**	1.0783	1.0324–1.1263
Sex (female)	−1.4943	**0.0079**	0.2244	0.0745–0.6757
PiO_2_/FiO_2_	−0.0089	0.0582	0.9911	0.9821–1.0003

**Table 5:** Stepwise logistic regression of demographic and clinical predictors of a positive disease outcome (discharge alive and/or NEWS2 ≤ 2 for at least 24 h within 14 days from hospital admission). The variables entered into the model are shown in the table. Sex (female) and NEWS2 score did not enter in the model. Bold indicates statistically significant values.

Parameter	Coefficient	*p*-Value	Odds Ratio	95% CI
Age	−0.0847	**<0.0001**	0.9188	0.8836–0.9555
25(OH)D	0.0469	**0.0494**	1.0481	1.0001–1.0983
PiO_2_/FiO_2_	0.0113	**0.0072**	1.0113	1.0031–1.0197

**Table 6:** Stepwise logistic regression of demographic and clinical predictors of a positive disease outcome (discharge alive and/or NEWS2 ≤ 2 for at least 24 h within 14 days from hospital admission) considering a 25(OH)D cut-off ≥ 11 ng/mL. The variables entered into the model are shown in the table. Sex (female) and NEWS2 score did not enter in the model. Bold indicates statistically significant values.

Parameter	Coefficient	*p*-Value	Odds Ratio	95% CI
Age	−0.0812	**<0.0001**	0.9220	0.8875–0.9579
25(OH)D	0.8971	**0.0445**	2.4524	1.0223–5.8832
PiO_2_/FiO_2_	0.0121	**0.0033**	1.0122	1.0040–1.0204

## Data Availability

Data will be made available from the corresponding author upon reasonable request.
